# Biomedical Progress Rates as New Parameters for Models of Economic Growth in Developed Countries

**DOI:** 10.3390/ijerph10115936

**Published:** 2013-11-08

**Authors:** Alex Zhavoronkov, Maria Litovchenko

**Affiliations:** 1 The Biogerontology Research Foundation, London W1J 5NE, UK; E-Mail: maria.litovchenko@bg-rf.org; 2 Moscow Institute of Physics and Technology, Dolgoprudny 141700, Russia; 3 Ludwig-Maximilians-Universität München, Munich 80539, Germany

**Keywords:** rejuvenation rate, biomedical advances, economic growth theory, retirement age, longevity

## Abstract

While the doubling of life expectancy in developed countries during the 20th century can be attributed mostly to decreases in child mortality, the trillions of dollars spent on biomedical research by governments, foundations and corporations over the past sixty years are also yielding longevity dividends in both working and retired population. Biomedical progress will likely increase the healthy productive lifespan and the number of years of government support in the old age. In this paper we introduce several new parameters that can be applied to established models of economic growth: the biomedical progress rate, the rate of clinical adoption and the rate of change in retirement age. The biomedical progress rate is comprised of the rejuvenation rate (extending the productive lifespan) and the non-rejuvenating rate (extending the lifespan beyond the age at which the net contribution to the economy becomes negative). While staying within the neoclassical economics framework and extending the overlapping generations (OLG) growth model and assumptions from the life cycle theory of saving behavior, we provide an example of the relations between these new parameters in the context of demographics, labor, households and the firm.

## 1. Introduction

Increases in productivity, technological progress and population growth have been identified as the main drivers of economic growth [1,2,3]. The endogenous growth theory expands on the underlying factors that affect technological advancement by focusing on human capital [4,5]. The unified economic theories further build on these ideas, incorporating elements of natural selection into growth models to explain an economy’s transition from stagnation to growth [6].

**Figure 1:**
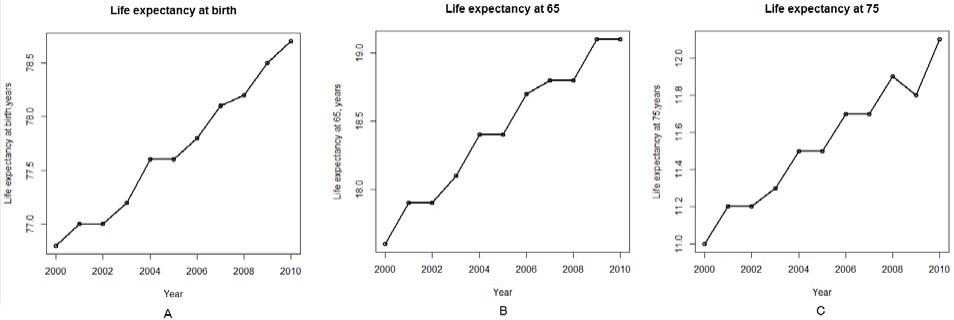
Life expectancy by age in the United States in the first decade of the twenty first century. (**A**) Life expectancy at birth. (**B**) Life expectancy at age 65. (**C**) Life expectancy at age 75. Data is provided by National Center for Health Statistics. Health, United States, 2012.

However, all of these models may be further extended to account for the rate and nature of technological advances, as well as for the lifespan, average years in the workforce, and the retirement age of the population. The past fifty years have seen more advances in science and technology than the rest of human history [7]. Concurrently, the world population has more than doubled, growing from 3 billion in 1960 to 7 billion in 2012 [8]. Life expectancies, meanwhile, have increased steadily across all segments of the population over the last twenty years [9]; Figure 1A–C; these figures were generated using R software using the data from National Center for Health Statistics, Health, United States, 2011 and 2012) [10,11]. These trends significantly increase the number of older people in the population and the length of time these people remain dependent on social security and healthcare systems after retirement. Recent studies have demonstrated that generational accounting may affect economic growth and must be included when evaluating the state of an economy [12]. Recent studies introduced the economic models for the notion of modifiable life spans and started raising the awareness of life spans rising beyond the “natural” improvements of life expectancy [13]. However, the effects of aging on economic growth remains a controversial topic in macroeconomics with some studies suggesting a positive relationship between longevity and growth due to the prevalence of the effects of capital accumulation [14] or shifts in labor force participation [15]. Studies from Japan proposed positive relationship between population aging and economic growth in a general equilibrium model of life cycle savings combined with endogenous growth and even suggested that postponing the retirement age will have a negative effect on economic growth [16]. 

Other theoretical models suggest the negative effect of aging on economic growth due to the increase in the dependence rate leading to reallocation of labor from non-health to health production [17]. Other studies using empirical evidence demonstrate the ambiguous effects of population aging on economic growth and suggest that in the economy, where the availability of capital is endogenously determined by domestic savings, the capital accumulation effect prevails and population aging leads to the economic growth [18].

Many technological advances occur at an exponential rate; for instance, Moore’s law, which states that computing power doubles every two years, has held for almost half a century [7,19]. However, in many areas of biomedicine, a field in which new discoveries can result in significant increases in the human lifespan, progress is much slower [20]. Innovations in information technology enter mainstream use rapidly, sometimes in a matter of months. Discoveries in the biomedical sciences, however, require thorough testing in laboratory animals and lengthy human trials, which delay the mass adoption of these innovations by years or even decades. As a result, life expectancies tend to grow slowly, increasing by just 2–3 years every decade. At this pace, it would take close to three hundred years for human life expectancy to double—a far cry from the rate of progress achieved by other disciplines.

Thus, the demographic effects of biomedical discoveries made after 1990, such as increases in lifespan and ability to perform useful work, will not be noticeable until at least 2030. Some promising 21st century advances that have the potential to increase life expectancy, like induced pluripotent stem cells and *in-vitro* organs made from endogenous patient cells, will not reach clinical practice for at least another decade [21,22]. The impact of recent advances in the biomedical sciences is also obscured by the detrimental effects of unhealthy lifestyle changes, such as easy and affordable access to high-calorie foods and a lack of physical activity, which apply negative pressure to the life expectancy curve.

### 1.1. Recent Acceleration of Biomedical Progress

In recent years, many areas of science and technology have experienced exponential growth in the number of scientists doing research in the field, funding and scientific publications. While many of the discoveries that have most dramatically transformed modern society have been concentrated in the field of information technology (for instance, cell phones, computers and the Internet), biomedical advances have accelerated at a similar pace. There have been more discoveries made in the biomedical field since 1960 than during the entirety of human history prior to 1960 [23]. The general paradigm of performing biomedical research and applying it to clinical practice involves seven major steps:

1. Conception of the idea
2. Describing the idea and applying for research grants
3. Performing experiments
4. Publishing the experimental results
5. Preclinical proof of efficacy and safety
6. Clinical trials
7. Mainstream clinical adoption

Scientific publications are an excellent indicator of biomedical progress. Medline, the centralized resource developed by the US National Library of Medicine, is used for indexing and tracking scientific publications. From 1993 to 2011, the number of scientific publications indexed in Medline increased from just over 400 thousand to just under one million papers per year [24]. In recent years, this number has grown even more dramatically as emerging countries increase their contributions to international science. 

Scientific grants, which measure future advances in biomedicine, may be an even better indicator of progress. Years or even decades may pass between the awarding of a grant and the publication of experimental results, but most funding institutions publish grant information after the grant is awarded. The resource developed by our team, the International Aging Research Portfolio (IARP), tracks the grant data available from major funding organizations in the US, Europe, Canada and Australia [25]. Annual science funding in the form of grants tracked by the IARP increased from just over $5 billion per year in 1993 to over $45 billion in 2011. This figure may be much more significant if other funding sources are accounted for. Similarly, private sector spending on R&D increased from over $35 billion in 1996 to over $95 billion in 2009 [26], with the top 15 pharmaceutical companies accounting for over $75 billion of the total. We expect this trend to continue in the coming years, fueling the number of publications, clinical trials and clinical procedures with the potential to extend life expectancy.

### 1.2. Longevity and Healthy Productive Lifespan

#### 1.2.1. Life Extension *vs.* Rejuvenation Rate

For the purposes of this model we assume that biomedical progress has a positive effect on life expectancy. Further, biomedical progress may be separated into two rates:

1. Rejuvenation rate—the sum of all developments extending a person’s ability to perform useful work and restoring function lost due to aging, disease or injury.
2. Non-rejuvenating biomedical progress rate—the sum of all developments that extend the lifespan, but do not extend the person’s ability to perform useful work or restore lost function.

**Figure 2:**
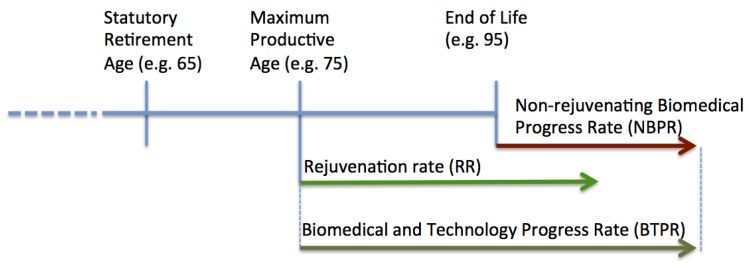
Rejuvenation Rate *vs*. Non-rejuvenating Biomedical Progress Rate. The figure depicts the hypothetical person’s lifespan, where the rate of biomedical and technology progress is comprised of the Rejuvenation Rate (RR) extending Maximum Productive Age and Non-rejuvenating Biomedical Progress Rate (NBPR) delaying the Age of Death, while not restoring the ability to do useful work.

Here we separate the rate of biomedical advances into two categories: the rejuvenation rate and non-rejuvenating biomedical progress rate. The concept is illustrated in Figure 2. Examples of biomedical developments extending the maximum productive age include preventative cancer vaccines, arthritis and diabetes treatments. Examples of non-rejuvenating developments include late-stage cancer, multiple sclerosis and other medications marginally extending patient’s life near end of life. 

The non-rejuvenating treatments are usually partially covered by insurance, but are expensive and in many cases exceed the family’s ability to afford the treatments. In 2007 in the US 61.2% of personal bankruptcies were healthcare-related [27]. In OECD countries the majority of healthcare expenditures are spent for the care of the terminally ill and costs increase with the age of the patients [28]. Population aging results in a rapid increase in healthcare expenditures [29,30]. A significant part of these expenses falls on the government thereby increasing public spending and fiscal deficits. This problem starts at the root source of the biomedical pipeline, where governments and companies focus on taking the treatments that extend life in terminally-ill patients because it is easier to conduct these studies and expedite the propagation of these treatments into clinical practice in the tight regulatory environment. Proving the efficacy of the preventative treatments may take many decades and the compensation mechanisms for these treatments are not clear. Using pattern analysis algorithms it may be possible to analyze the current research clinical trials pipelines and re-focus the research activities to accelerate the rejuvenation rate. 

## 2. Model

Our model is based on the OG model and on the life-cycle theory of saving behavior [31]. The economy consists of two types of agents: households and the firm. We incorporate three new features into the model:

(i) Rejuvenation rate (RR)—the rate at which the functions required to perform useful work that were lost to aging or disease are restored
(ii) Non-rejuvenating rate of biotechnical progress (NRPR)—the rate of biotechnical progress that increases lifespan, but does not return lost functions
(iii) Biomedical science and technology progress rate (BMTPR)—the rate at which progress in science and technology and the application of these technologies to clinical practice extends the lifespan of the population. It is equal to RR + NRPR

Additional parameters introduced into the OLG model are summarized in Table 1 and the list of basic functions is summarized in Table 2.

**Table 1:** Basic Parameters.

Designation	Description	Comment
A	*Age*	
t	*Time*	
R	*Rejuvenation* Partial or complete restoration of the ability to perform useful work lost due to illness, injury or aging	
RR	*Rejuvenation rate* The rate at which advances in technology and the natural, social and behavioral sciences and the mainstream adoption of these advances lead to the partial or compete restoration of the ability to perform useful work lost to illness, injury or aging. Example: advances in arthritis treatment and prevention, procedures that restore vision, software products assisting with memory loss.	
S	*Retirement age* The age set by the government at which the individual is entitled to receive full social security and healthcare benefits	
NRPR	*Non-rejuvenating progress rate* Advances in science and technology and the mainstream adoption of these advances that extend lifespan but does not restore the ability to perform useful work. Example: drugs and therapies that increase survival of terminally ill patients by several months, but do not restore healthy function and the ability to perform useful work.	The rate of biotechnical progress that increases lifespan, but doesn’t return lost functions.
BMTPR	*Biomedical science and technology progress rate* The rate at which progress in science and technology and the application of these technologies to medical practice extends the lifespan of the population. It is equal to RR+NRPR	
A_aver_	*The average lifespan*	
MPA	*Maximum productive age* The age at which the ability to do useful work is diminished. The age may be increased through rejuvenation (R).	
A_max_	*Maximum lifespan*	

**Table 2:** Basic functions.

Designation	Description	Comment
N (A,t, BMTPR)	The number of people of age A in period t	
Pr (A,t,RR, BMTPR)	The productivity of labor	
W_r_ (A,t, BMTPR )	The ratio of the working population of age (A) to the total population of age (A)	
В (RR)	Biological retirement age	The age at which a person can no longer work due to biological reasons, *i.e.*, illness.
L_s_(RR)	The percentage of people that will retire at age S	
M_r_ (A,BMTPR)	Mortality rate	0 < M_r_ < 1 The probability of dying at age A
Br(t)	Birth rate	The ratio of the number of newborns in period t to the total number of people in the population
AdRate(t)	The mainstream clinical adoption rate of biomedical discoveries	The rate of adoption of biomedical discoveries by hospitals and doctors. 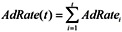

Households are assumed to be rational, production technology is given by a standard Cobb-Douglas function, and the one-good economy is assumed to be closed. Bequest motives are not included [31].

### 2.1. Demographics

The economy consists of people, aged from 0 to A_max_, denoted by i = 0; 1; 2; …; A_max_. In each new period t, a new generation aged 0 is born into the economy, while each of the existing generations shifts forward by one. The oldest generation, i = A_max_, which we assume to be the maximum age, dies out. 

We assume that each age is characterized by a mortality rate—the percentage of people of that age that will die. The mortality rate is described by the function:

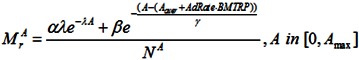
(1)
where A_aver_—average lifespan; α, β and γ—parameters for normalizing the mortality rate, N^A^—number of people at the age A. 

**Figure 3:**
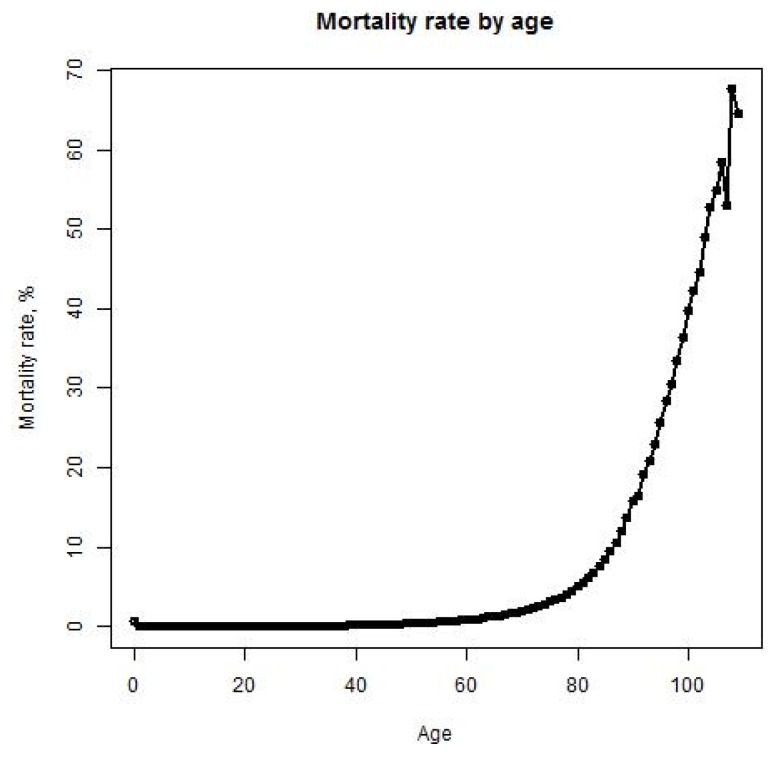
Mortality rate in the United States in 2010. The X-axis represents age and the Y-axis represents the mortality rate in percent. The mortality rate is calculated as the ratio of the number of deaths among people of a certain age to the total number of people of a certain age.

This distribution takes into consideration the correlation between mortality rate and age, based on the statistical data provided by mortality.org (See Figure 3). It also allows people to achieve maximum age.

Because it can take many years for medical professionals to adopt a life-prolonging biomedical discovery, the mortality rate formula includes the mainstream clinical adoption rate of these discoveries (AdRate):

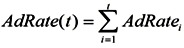
(2)

According to the above formula, the adoption rate of a biomedical discovery in year t is the sum of adoption rates from year 1, when the discovery is made, to year t. The adoption rate for a certain year can be characterized as the percentage of medical institutions in the country that start using the discovery during that year. For instance, if 2% of all medical institutions begin using a new biomedical procedure in the first year after its discovery, and a further 10% begin using it in the subsequent year, the AdRate for year 2 would be 12%. Of course, AdRate is a function and could change from period to period. Also, AdRate cannot, by definition, exceed 1. 

Combining all of these these parameters, the number of people living in the economy at the beginning of period t + 1 is calculated as:

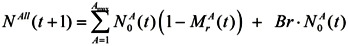
(3)

### 2.2. Employment and Labor

**Figure 4:**
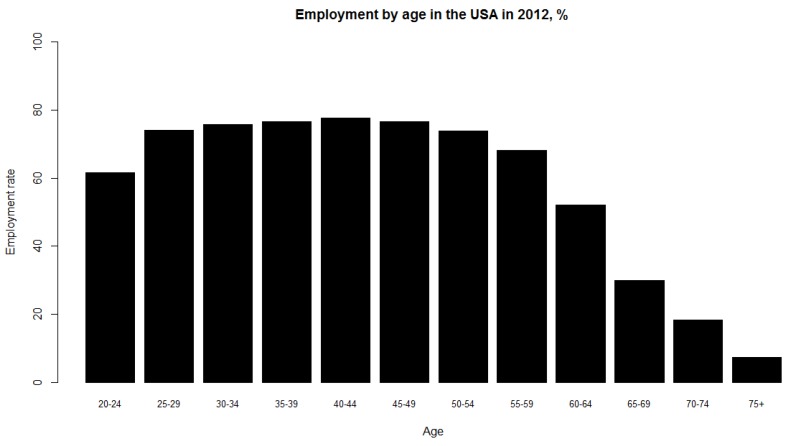
Employment rate by age group in the United States in 2012. The X-axis represents age and the Y-axis represents the employment rate, calculated as the ratio of employed population in the labor force to total population in the labor force.

(1) We assume that the percentage of people of age A in the labor force is described by the function:

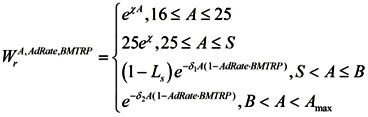
(4)
where S the age set by the government at which the individual is entitled to receive full social security and healthcare benefits B which denotesthe age at which a person can no longer work due to biological reasons, *i.e.*, illness, χ—proportion of the population that enters the labor force between 16 and 25 years of age. This parameter is introduced since people do not enter the labor force all at once; rather, labor force participation increases steadily between ages 16 and 25 (See Figure 4).

δ_1_—the proportion of the population that will exit the labor force after the retirement age set by the government (S) and before the biological retirement age (B). This parameter is introduced since people do not exit the labor force at the same time. This paradigm may change as a result of shifts in the retirement culture—for instance, more people may choose to work until their biological capacity is exhausted. Moreover, it has been demonstrated that a notable proportion of older workers can perform tasks as competently as their younger counterparts [32,33].

δ1 << 1
(5)

δ_2_—the proportion of the population that will exit the labor force after the biological retirement age. This parameter is introduced since some people will continue working throughout their entire lives, even after exhausting most of their biological capacity for work. We assume that after reaching the biological retirement age, people exit the labor force faster than they do following the retirement age set by the government.

δ2 > δ1
(6)

This distribution takes into account that people under age 16 are not working.

S—the retirement age set by the government. At this age, a person can either decide to retire or continue working. Denote by P the percentage of people who choose not to retire upon reaching retirement age.

B—the biological retirement age. At the biological retirement age and beyond, a person’s capacity to perform work effectively diminishes as a result of health issues. 

(2) We assume that the productivity of workers is not constant and depends on age, and is described by a Gaussian distribution. This model is based on the work of Lee and Yaari [34,35] and takes into account the finding that older workers can perform tasks as effectively as their younger counterparts:

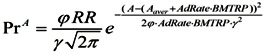
(7)
where φ = const—the parameter that takes into account the correlation between RR and Pr.

(3) So the total supply of labor available in the economy is calculated as:

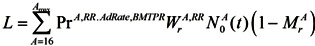
(8)

### 2.3. Households

Until retirement, households supply labor to the firm and earn wage (w) income according to their age-specific labor efficiency.

Although studies modeling consumer behavior in light of uncertain lifetime exist [36], here we assumed only the representative households to estimate the lifetime utility. Households aged j > S start to withdraw from the labor force, and receive public pension benefits from the social security system. Households own capital and rent it to firms throughout their lives. They consume the rest of their income [34]. 

Households maximize their expected lifetime utility (discounted by the probability of dying in the next period):

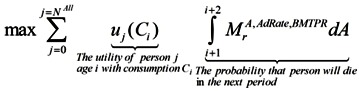
(9)
Subject to the budget constraints over their lifetime:

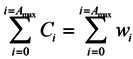
(10)

### 2.4. Firm

There is a representative firm producing final goods with the Cobb-Douglas production technology. In perfectly competitive spot-markets, the firm rents capital and hires labor from households so as to maximize its profit:

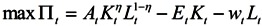
(11)
where K_t_ is aggregate capital stock, L_t_ is aggregate labor input, and R_t_ is the rental rate of private capital. A_t_ denotes the total factor productivity (hereafter TFP) in period t, and we assume that the TFP grows at the rate of g_t_ in every period.

### 2.5. Competitive Equilibrium

A competitive equilibrium consists of: 

Households maximizing their lifetime utility:

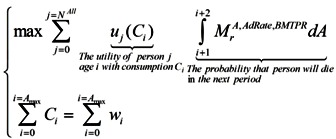
(12)
and the firm maximizing its profit:

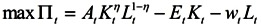
(13)
so that:

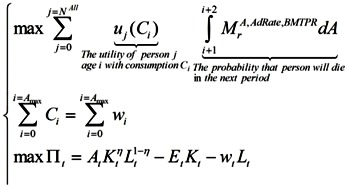
(14)

Also we assume all markets clear, *i.e.*, for all t, we have L_t_ = 1, b_t_ = 0, and c_1,t_ + c_2,t_ + k_t_ + 1 = F(k_t_, 1), where c_1,t_ be worker t’s consumption when young, and let c_2,t+1_ be his consumption when old, k_t_ is the t’s capital and F(k_t_, 1)—neoclassical production function [37].

### 2.6. Sensitivity Analysis to BMTPR

BMTPR will have a positive effect on lifespan, productivity and biological retirement age, but its effect on the percentage of the population in the labor force depends on the RR/BMTPR ratio. 

**Figure 5:**
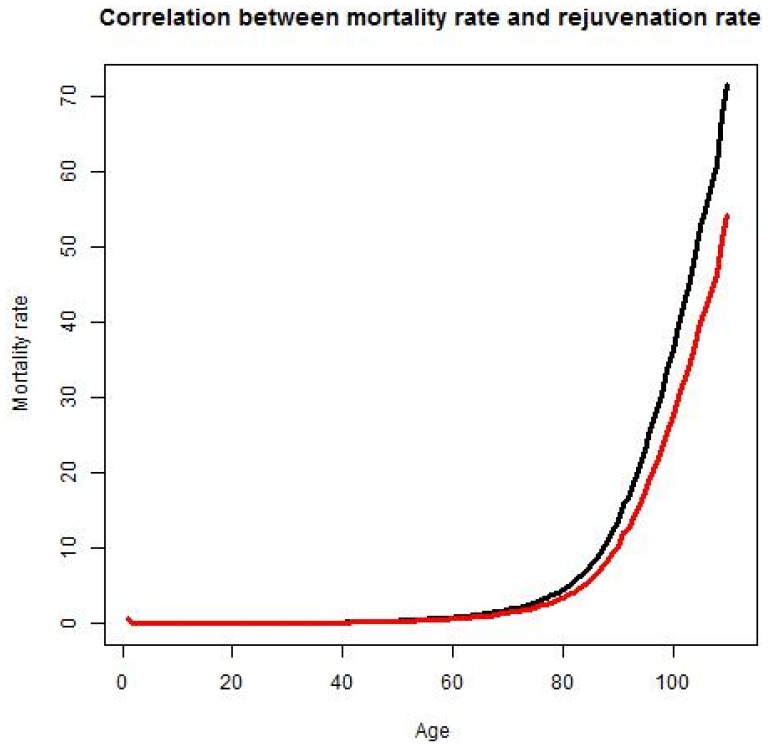
Mortality and rejuvenation rates. Mortality rate at age A is the percentage of people of age A that will die at age A. Rejuvenation rate is the rate at which advances in science and technology and the application of these advances to clinical practice lead to the partial or complete restoration of the ability to perform useful work lost to illness, injury or aging.

When RR increases, the mortality rate will peak at a later age, decreasing M_r_ and increasing the lifespan (Figure 5):

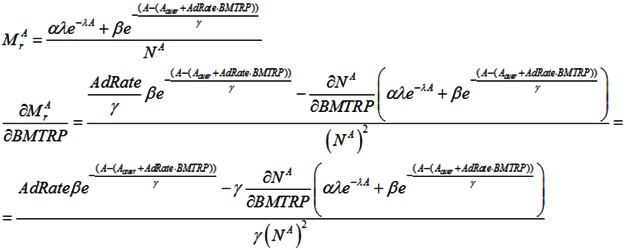
(15)

BMTPR will increase the biological age of retirement. The relationship between these two factors is likely to be linear. 

B = 60p·(1 + AdRate·BMTRP), where p is a parameter that takes into account the correlation between RR and B. 

**Figure 6:**
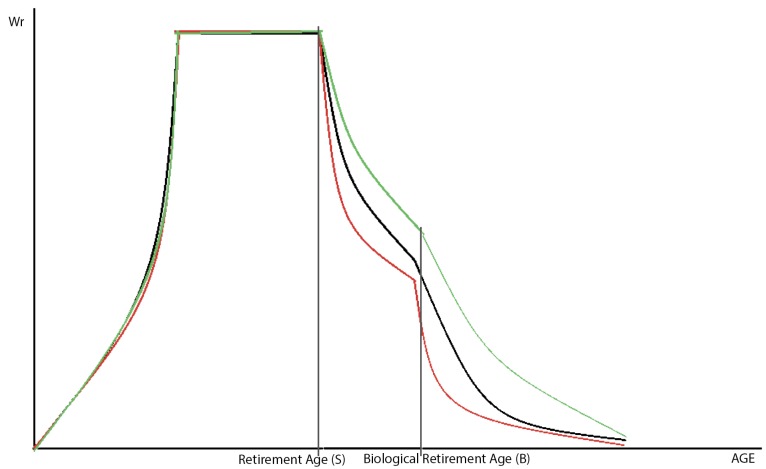
Effects of the rejuvenation rate on labor participation. Employment rate without BMTPR (BMTPR = 0) is represented by the black curve, with BMTPR (BMTPR ≠ 0) and RR/BMTPR << 1/2 is represented by red curve, with BMTPR (BMTPR ≠ 0) and RR/BMTPR >> 1/2 is represented by the green curve. The X-axis represents age.

By improving the quality of life, BMPTR will increase the time people spend in the labor force (Figure 6). Thus, the number of people who do not retire at age S will increase. L_s_ = L_s_^0^(1–RR)·p_2_, where p_2_ is a parameter that takes into account the correlation between RR and L_s_.

Statistics show that increases in a country’s average life expectancy are correlated with decreases in the birth rate, so we can conclude that an increase in RR will lead to decrease in birth rate.

**Figure 7:**
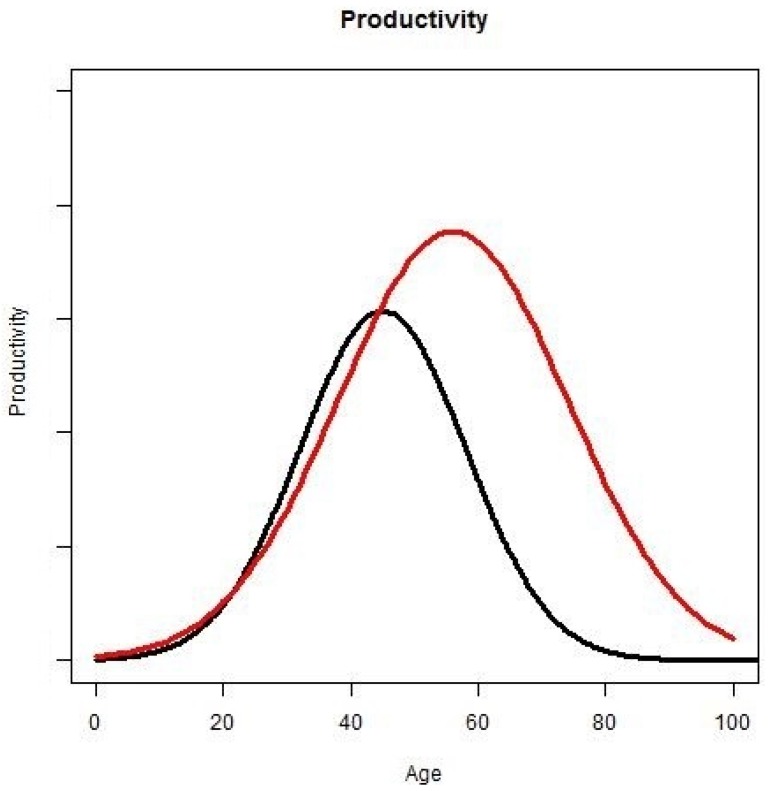
Productivity and rejuvenation rate. The X-axis represents age and the Y-axis represents productivity. The black curve illustrates the relative productivity with age without rejuvenation rate. The red curve illustrates the relative productivity with age with rejuvenation rate.

Workers’ productivity depends on their age (Figure 7); for instance, the productivity of teenagers is lower than the productivity of people in their thirties. Productivity also depends on the rejuvenation rate. With more active years, people have more time for education, thus increasing their productivity:

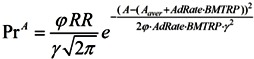
(16)

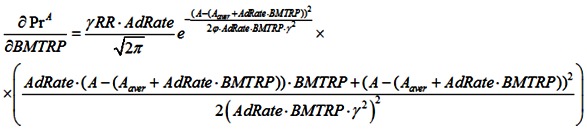
(17)

### 2.7. The Difference between the Effects of RR and BMTPR on the Economy

It is worthwhile to note the difference between the effects of RR and NBPR. In particular, the rejuvenation rate increases the number of people who are able to work, while the non-rejuvenating progress rate increases the number of people who are unable to work. Thus, the effect of BMTPR on an economy depends on the RR/BMTPR ratio. For example, a RR/BMTPR ratio of less than 1 would result in a shortage of workers and a consequent decrease in economic growth.

Most medical advances today extend the lifespan of sick people in the last months or years of their life without enabling them to make productive contributions to the economy. In the developed countries the majority of the lifetime healthcare costs are attributed to the last years of a patient’s life and the cost of healthcare is rapidly increasing with age in late life [37,38]. For example, new cancer drugs may extend cancer patients’ lives by several months compared to standard methods of care, but are significantly more expensive. The approval and clinical introduction of drugs like Dendreon’s Provenge vaccine have already triggered broad public cost/benefit debates [39]. Note that while a person remains unable to work, he or she does not contribute to GDP. Moreover, the cost of maintaining his or her life reduces GDP, even as the overall mortality of population declines:

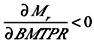
(18)

This leads us to the conclusion that it is the RR/BMTPR ratio that affects the economy, rather than the RR or BMTPR in particular. Thus, biotechnical progress will lead to economic growth only if

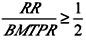
(19)

because if mortality decreases faster than the ability to work and the retirement age increase, the economy must maintain more pensioners. In contrast, by increasing the period of active life, we give a person the opportunity to work longer; as a result, fewer people reaching the government retirement age S will retire, and the rate of withdrawal from the labor force (α) is also reduced.

To sum up the effects of the RR/BMTPR ratio on the economy, we analyze its influence on labor, which will lead to direct influence on GDP:

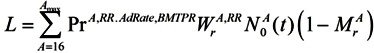
(20)

If the RR/BMTPR ratio increases, such components of labor as Pr^A,RR^, W_r_^A,AdRate,BMTRP^, (1−M_r_^A^) will increase, but N_0_^A^(t) will decrease. 

So an increase in the RR/BMTPR ratio could lead to the problem of unemployment:

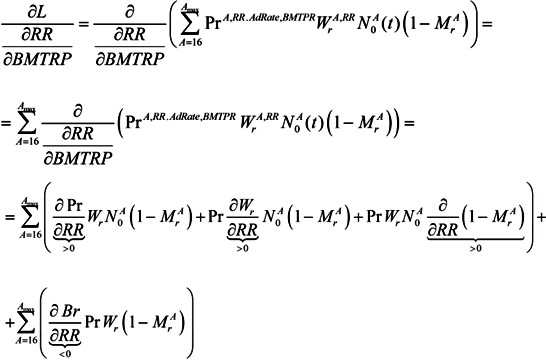
(21)

The problem of unemployment will not occur if

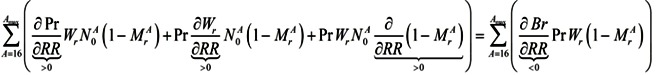
(22)

This implies that birth rate must decrease quicker than the number of people who are able to work increases. Statistics show that the speed of birth rate reduction accompanying increases in lifespan is very quick, which suggests that the economy would not face increasing unemployment in this scenario.

As long as GDP in our model is strictly increasing with increase of labor, GDP will increase with RR/BMTPR ratio.

## 3. Conclusions

In this paper, we have introduced several novel parameters that may be incorporated into the most commonly accepted theories of economic growth in addition to frequently cited factors like population growth, technology, behavior, capital flows, and distribution of capital. These parameters are a very recent phenomenon and can only be traced back for the past two decades. We propose a model that takes into account progress in the biomedical sciences, which in turn affects the size, growth and productivity of the population. In the model, the rate of biomedical progress is the sum of the rejuvenation rate, the rate at which the functions required to perform useful work that were lost to aging or disease are restored, and non-rejuvenating rate, which increases lifespan, but does not restore lost functions. 

We hypothesize that, over the past two decades, economic growth in the developed countries has been partially defined by the ratio of the rejuvenation rate to the overall biomedical progress rate and the retirement age. The biomedical progress rate extends the lifespan and decreases the mortality rates of the population, while the rejuvenation rate allows for the increased productivity of older workers and increases in the retirement age. 

We propose that the increase in the ratio of the rejuvenation rate to the overall biomedical progress rate will result in economic growth. This hypothesis is supported by recent studies showing that the acceleration of aging research focused on increasing longevity and postponing age-related diseases and not the treatment of age-related diseases [40]. Another source of economic growth may come from accelerating the rate of clinical adoption by reducing the time it takes for a biomedical discovery to reach the patient.

The effects of population aging on economic growth remains a controversial topic in macroeconomics with conflicting schools of thought. While there are many models and simulations that account for population aging [41,42,43], the new parameters introduced in this paper may help enrich the models demonstrating both positive and negative effects of aging on the economy and help model scenarios that go beyond extending historic trends in longevity.
